# Suture Fiber Reinforcement of a 3D Printed Gelatin Scaffold for Its Potential Application in Soft Tissue Engineering

**DOI:** 10.3390/ijms222111600

**Published:** 2021-10-27

**Authors:** Dong Jin Choi, Kyoung Choi, Sang Jun Park, Young-Jin Kim, Seok Chung, Chun-Ho Kim

**Affiliations:** 1 Laboratory of Tissue Engineering, Korea Institute of Radiological and Medical Sciences, 75, Nowon-ro, Nowon-gu, Seoul 01812, Korea; choidj@kirams.re.kr (D.J.C.); choik1231@kirams.re.kr (K.C.); sjpark@kirams.re.kr (S.J.P.); 2 Program in Biomicro System Technology, Korea University, Innovation Hall, 145, Anam-ro, Seongbuk-gu, Seoul 02841, Korea; sidchung@korea.ac.kr; 3 Department of Biomedical Engineering, Catholic University of Daegu, 13-13, Hayang-ro, Hayang-eup, Gyeongsan-si 38430, Korea; yjkim@cu.ac.kr

**Keywords:** 3D printing, gelatin biomaterial ink, suture fiber, 3D scaffold, tissue engineering

## Abstract

Gelatin has excellent biological properties, but its poor physical properties are a major obstacle to its use as a biomaterial ink. These disadvantages not only worsen the printability of gelatin biomaterial ink, but also reduce the dimensional stability of its 3D scaffolds and limit its application in the tissue engineering field. Herein, biodegradable suture fibers were added into a gelatin biomaterial ink to improve the printability, mechanical strength, and dimensional stability of the 3D printed scaffolds. The suture fiber reinforced gelatin 3D scaffolds were fabricated using the thermo-responsive properties of gelatin under optimized 3D printing conditions (−10 °C cryogenic plate, 40–80 kPa pneumatic pressure, and 9 mm/s printing speed), and were crosslinked using EDC/NHS to maintain their 3D structures. Scanning electron microscopy images revealed that the morphologies of the 3D printed scaffolds maintained their 3D structure after crosslinking. The addition of 0.5% (*w*/*v*) of suture fibers increased the printing accuracy of the 3D printed scaffolds to 97%. The suture fibers also increased the mechanical strength of the 3D printed scaffolds by up to 6-fold, and the degradation rate could be controlled by the suture fiber content. In in vitro cell studies, DNA assay results showed that human dermal fibroblasts’ proliferation rate of a 3D printed scaffold containing 0.5% suture fiber was 10% higher than that of a 3D printed scaffold without suture fibers after 14 days of culture. Interestingly, the supplement of suture fibers into gelatin biomaterial ink was able to minimize the cell-mediated contraction of the cell cultured 3D scaffolds over the cell culture period. These results show that advanced biomaterial inks can be developed by supplementing biodegradable fibers to improve the poor physical properties of natural polymer-based biomaterial inks.

## 1. Introduction

Tissue engineering scaffolds are widely used not only for tissue regeneration, but also for disease models, drug screening, and cell (or drug) delivery systems [1,2,3]. In order to fabricate tissue engineering scaffolds for the desired application, a sophisticated fabrication technology is required. Additive manufacturing technologies use machine and computer-aided design to fabricate a 3D structure by specifically carrying out a layer-by-layer deposition of a biomaterial [4,5]. Among them, extrusion-based 3D printing technology has been actively researched in the tissue engineering field for the past few decades because it can provide possibilities for conveniently fabricating complex 3D biomimetic structures using a combination of various types of cells and biomaterials [6]. These complex 3D printed biomimetic scaffolds reproduce not only the structural and constitutive properties of human tissues, but also their biological properties. In order to obtain such a 3D scaffold, the pre-printing, 3D printing, and post-printing processes must be optimized [7]. The pre-printing process involves selecting the material to be used as the biomaterial ink. In the 3D printing process, 3D printing conditions are optimized and 3D scaffolds are fabricated under these conditions. Moreover, the post-printing process involves evaluating the physicochemical and biological properties of the 3D printed scaffold. It is impossible to overemphasize the importance of these processes. These processes begin with selecting an appropriate biomaterial for the biomaterial ink [8].

Biomaterial ink, one of the major components of 3D printed scaffolds, has several issues in selecting material [9]. Physically, biomaterial ink materials should have sufficiently good rheological properties to be 3D printed as continuous strands, and they must have appropriate mechanical strength for tissue engineering applications [10]. Biologically, it should have excellent biocompatibility for in vivo transplantation. In order to satisfy these issues, the concept of a “biofabrication window” was introduced [11]. Based on this biofabrication window concept (for improving the physical and biological properties of biomaterial ink), various natural polymer biomaterial inks have been studied [12,13,14,15]. Gelatin, one of these natural polymers, is a powerful candidate material for biomaterial ink because it has a chemical structure and biological properties resembling those of collagen and includes a cell adhesion motif (Arg-Gly-Asp) [16,17]. In addition, gelatin’s excellent biological properties such as high biocompatibility and biodegradability have already been demonstrated in many studies [18]. Despite these excellent biological properties, it is uncommon for pure gelatin to be used as biomaterial ink due to the poor rheological properties of gelatin [7]. Most studies have used modified gelatin bioink or multi-biomaterial ink in which other polymeric materials are added to gelatin [19,20,21,22]. As previous studies have shown, the low viscosity of pure gelatin can be sufficiently overcome by using the thermo-responsive behavior of the gelatin [23]. However, there is still a need for efforts to enhance the low mechanical strength of pure gelatin hydrogels and to improve the dimensional stability of the pure gelatin 3D scaffold during the cell culture period.

The mechanical strength and dimensional stability of the 3D scaffold directly affect tissue regeneration. The stiffness of human tissue varies greatly from Pa to GPa depending on the type of organ [24,25]. Implanted scaffolds that do not have adequate mechanical strength cannot maintain dimensional stability due to human tissue movement and external stimuli within the in vivo environment. Therefore, the 3D printed scaffold must have adequate mechanical strength for the applied organ. Pure gelatin biomaterial ink has low mechanical strength and high solubility in aqueous solutions [26]. Thus, crosslinking processes are necessary to improve the mechanical strength and dimensional stability of the gelatin hydrogel [27]. A simple way to crosslink gelatin is to use a chemical crosslinking agent such as 1-ethyl-3-(3-dimethylaminopropyl)-carbodiimide (EDC)/N-hydroxysuccinimide (NHS), an aldehyde, or genipin [28,29,30]. When deploying the chemical crosslinking method, the hydrogel mechanical strength can be controlled by changing the concentration of the crosslinking agent and the reaction time. According to H. Lv et al., the stiffness range of gelatin hydrogel crosslinked by EDC is 0.6–2.5 kPa [31]. However, while high concentrations of chemical crosslinking agents improve the mechanical strength of the gelatin hydrogel, they can cause cytotoxicity by unreacted chemicals remaining in the hydrogel [32]. In another method that has been investigated, various types of micro/nano particles are added into the biomaterial ink. According to X. Sun et al., when silica nanoparticles were introduced into gelatin-based biomaterial inks (gelatin methacryloyl (GelMA)/gelatin/4-arm poly (ethylene glycol) acrylate (PEG)), the 3D scaffold mechanical strength increased about 2-fold [33]. Despite these studies, it has been reported that fiber types are superior in improving the mechanical strength of 3D scaffolds over particle types. J. Zhang et al. confirmed that the mechanical strength of the chitosan 3D scaffold introduced with silk fiber was higher than that of the silk nanoparticles added 3D scaffold [34]. Another study, on comparative mechanical reinforcement of cellulose nanofibrils and cellulose nanocrystals, showed similar results [35]. The better results for fibers than for particles may be attributed to the higher aspect ratios of fibers better improving the mechanical strength of the 3D scaffold. For this reason, L. Huang et al. used cellulose nanofibers as an additional material in silk fibroin/gelatin biomaterial ink formulations for 3D printing and fabricated a 3D scaffold with a modulus of 186.5 kPa [36]. However, cellulose has the disadvantage of displaying poor biodegradability in the human body. For this reason, further studies are needed to increase the mechanical strength of pure gelatin biomaterial inks.

In this study, a biodegradable suture fiber (synthetic, absorbable, sterile surgical suture composed of a copolymer made of 90% glycolide and 10% L-lactide) was added into a pure gelatin biomaterial ink to improve the mechanical strength and dimensional stability of the 3D scaffold. The effects of different content of added suture fibers on the biomaterial ink rheological properties, printability, and printing accuracy were evaluated. Subsequently, we studied the physicochemical properties of the 3D printed scaffolds, including their mechanical strength, swelling ratio, and degradation properties. The cell affinities of the 3D printed scaffolds prepared with the addition of different contents of suture fibers were evaluated by culturing human dermal fibroblasts on each 3D printed scaffold. Finally, the improvement resulting from the suture fibers was demonstrated by examining the cell-mediated contraction and mechanical strength of the 3D printed scaffolds during the cell culture period.

## 2. Results and Discussion

### 2.1. Rheological Properties of Suture Fibers Added Biomaterial Ink

**Figure 1:**
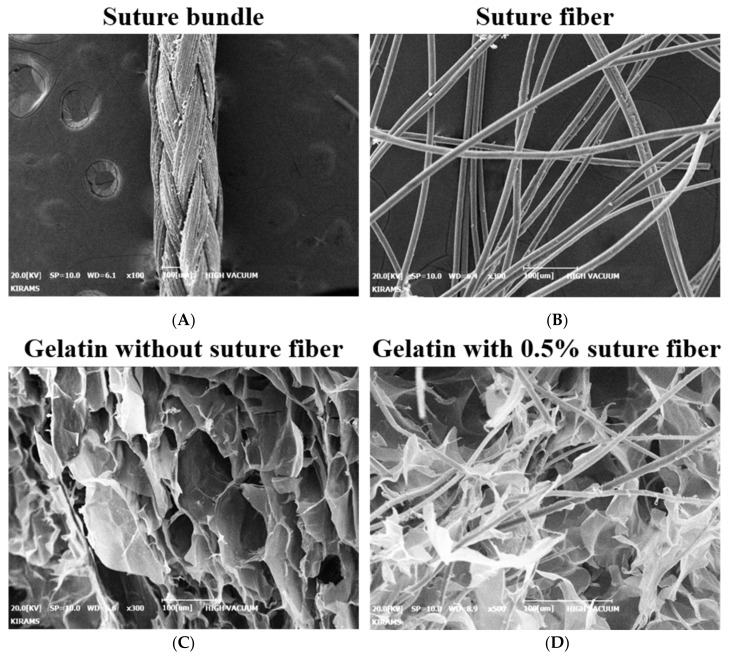
The SEM images of suture (bundle/fibers) and suture fibers added gelatin biomaterial ink. Suture (**A**) bundle and (**B**) fiber. Gelatin biomaterial ink (**C**) without suture fibers and (**D**) with suture fibers.

The SEM image shows that several micro-sized suture fibers are well contained in the gelatin biomaterial ink (Figure 1A–D).

**Figure 2:**
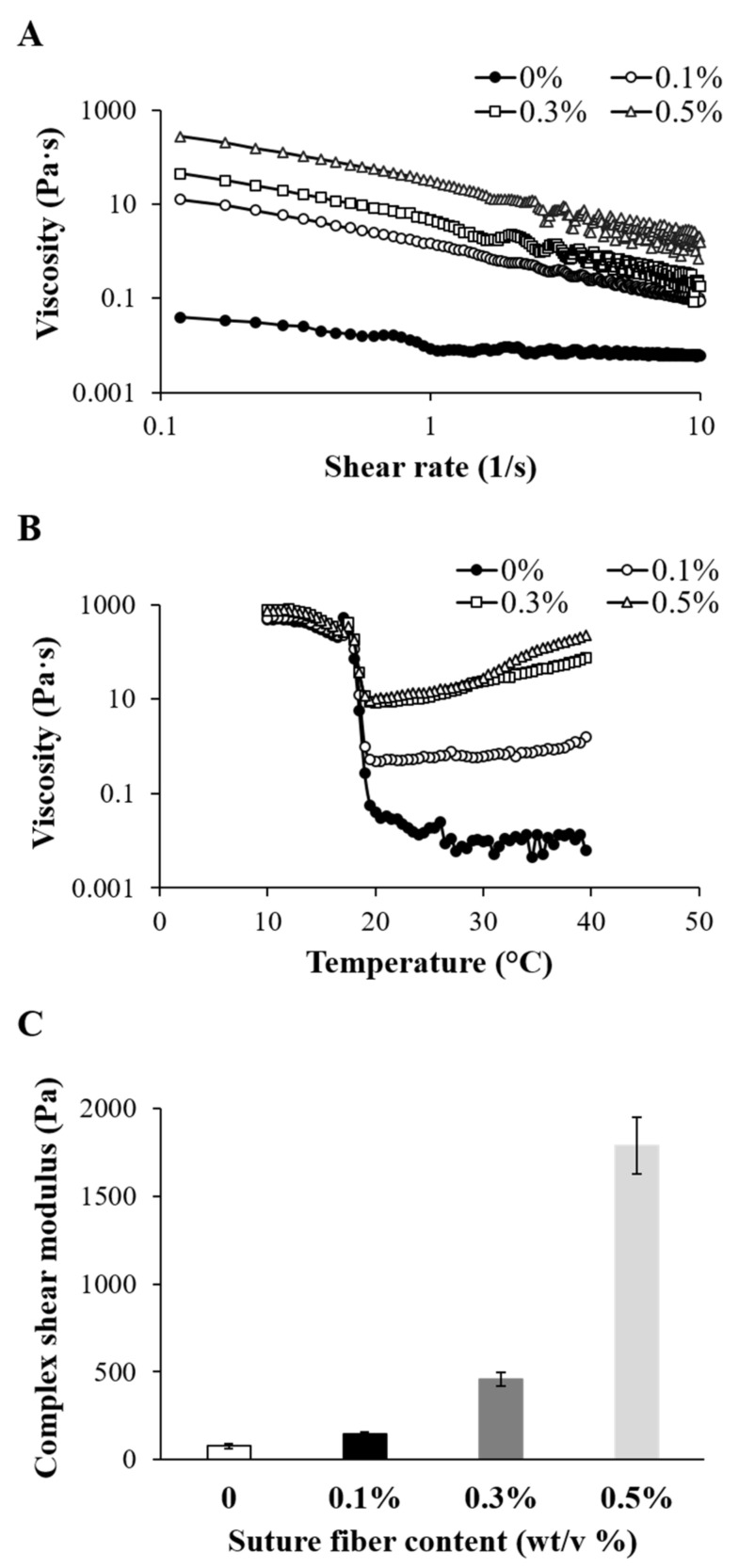
Rheological properties of each of the four different biomaterial ink formulations produced in the current work. (**A**) Viscosity as a function of shear rate. (**B**) Viscosity as a function of temperature. (**C**) Complex shear modulus measured at 1 Hz frequency (*n* = 4).

The rheological properties of a biomaterial ink, such as viscosity and gel strength, are very important factors in determining the printability and printing accuracy of the biomaterial ink [37]. In terms of rheological properties, gelatin biomaterial inks with low viscosity are not suitable for 3D printing. Therefore, we used the thermo-responsive behavior of gelatin to increase their viscosity. As previously shown, optimal rheological properties for gelatin biomaterial inks were evaluated [23]. Herein, it was necessary to confirm the effect of suture fibers on the thermo-responsive behavior of gelatin. A rheometer was used to determine the viscosity, gel point, and complex shear modulus of the suture fibers added gelatin biomaterial ink. As shown in Figure 2A, all the biomaterial inks showed obvious shear thinning behavior, which is an essential property for hydrogel-based biomaterial ink printing [38]. The flow curve of the biomaterial ink varied depending on the suture fiber content. The biomaterial ink viscosity increased from 0.01 Pa·s to 33.3 Pa·s as the suture fiber content was increased from 0% to 0.5% (at a 1/s shear rate). Notably, the viscosity of the biomaterial ink with 0.5% suture fiber content was about 3100 times higher than that of the biomaterial ink without suture fibers. This difference was due to the added suture fibers having absorbed moisture from the gelatin aqueous solution and the suture fibers having physically interacted with each other. When the suture fiber content was more than 0.5%, the fibers did not disperse homogeneously in the gelatin aqueous solution and instead became entangled. We were thus unable to implement a further increase in the suture fiber content to achieve a higher viscosity. In order to increase the viscosity of the suture fibers added gelatin biomaterial ink, we instead used the thermo-responsive behavior of the gelatin. As shown in Figure 2B, significant changes in biomaterial ink viscosity occurred when changing the temperature. The viscosity of the gelatin biomaterial ink increased rapidly as temperature was cooled to below 20 °C. Below 20 °C, the gelatin generated phase transition from spread chain to triple helix structure and this transition was reversible [39]. Adding suture fibers did not result in any significant change in the sol-gel transition temperature (18~20 °C). This result indicated that the sol-gel transition temperature of the gelatin biomaterial ink into which suture fibers were added was highly dependent on the concentration of the gelatin [23] but not dependent on the content of the added suture fibers. Based on these data, the temperature of the gelatin biomaterial ink reservoir should be kept at 20 °C during the 3D printing process. In the gel state biomaterial ink, the gel strength is an important rheological property in determining printability. As shown in Figure 2C, the complex shear modulus increased with increasing suture fiber content. The complex shear modulus of the 0, 0.1, 0.3, and 0.5% biomaterial inks were 77.4 ± 12.9, 146.4 ± 13.6, 459.7 ± 40.0, and 1789.5 ± 163.3 Pa, respectively. These results showed that the addition of suture fibers did not affect the sol-gel transition temperature but did affect the gel strength of the gelatin biomaterial ink. These results, in particular those showing an improvement in biomaterial ink rheological properties with the addition of micro/nano fibers, were consistent with those of several studies [34,36].

### 2.2. Printability of Suture Fibers Added Biomaterial Ink

**Figure 3:**
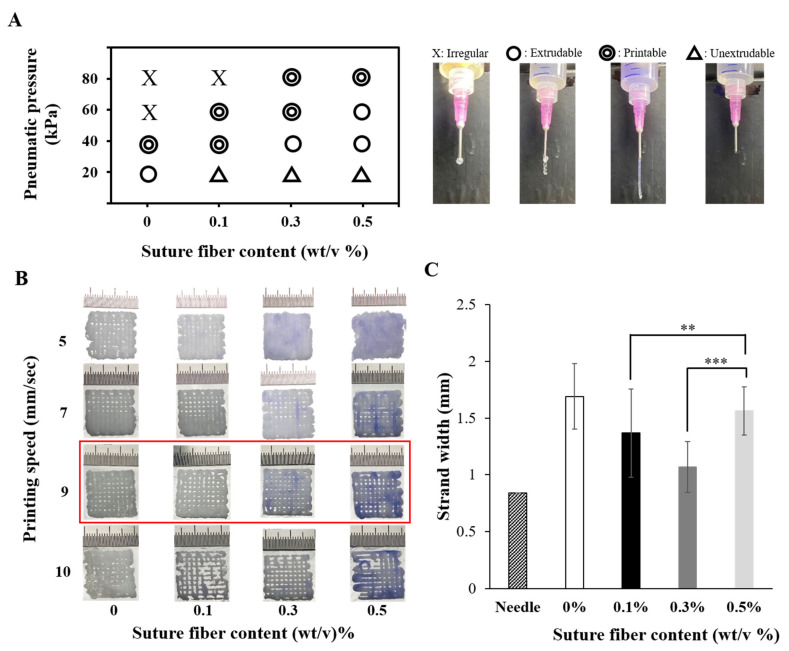
Printability of the biomaterial inks into which suture fibers were added. (**A**) Filament drop test results for determining the proper pneumatic pressure to be used. (**B**) Images of 3D scaffolds fabricated at various printing speeds. (**C**) Printed strand widths measured using vernier calipers. The strand width of the needle indicates the inner diameter of the 18-gauge needle (0.84 mm) used for printing (*n* = 6) (** *p* < 0.01 and *** *p* < 0.001).

G. Gillispie et al. noted that the word ‘printability’ was recently adopted in the 3D printing field to describe the capabilities of materials used in various 3D printing modalities [40]. Many conditions, such as biomaterial ink rheological properties, printing speed, pneumatic pressure, and temperature, are involved in determining printability. We have already determined certain rheological properties (sol-gel transition temperatures, gel strengths) of the gelatin biomaterial inks into which suture fibers were added (in Figure 2C), so we had to find a suitable pneumatic pressure and printing speed for each biomaterial ink. Figure 3A shows the extruded strand state for each of the various pneumatic pressures and suture fiber contents. When the pneumatic pressure was too low, the biomaterial ink was not able to come out through the nozzle (Δ: unextrudable in the figure). On the other hand, using too high of a pneumatic pressure yielded irregular strands (X: irregular). We found a suitable pneumatic pressure between 40 and 80 kPa for each biomaterial ink formulation made in the current work. As the suture fiber content in the biomaterial ink was increased, the ideal pneumatic pressure to print also increased. Note that these results were consistent with the rheological properties of the biomaterial ink (Figure 2C). As shown in Figure 3B, when the printing speed was less than 8 mm/s, irregular strands were produced and the printing time was too long (>10 min). On the other hand, when the printing speed was more than 10 mm/s, it was impossible to fabricate a 3D structure due to the break of the strands. Therefore, the appropriate printing speed, applicable to all four biomaterial inks, was determined to be 9 mm/s. Figure 3C shows the printed strand widths of the different biomaterial inks at their respective optimized pneumatic pressures. It was found that suture fibers added biomaterial ink (0.1% and 0.3%) could obviously reduce the printed strand width compared with the biomaterial ink without suture fibers. When suture fiber content was increased to 0.5%, the strand width was similar to that of the biomaterial ink without suture fibers (1.69 ± 0.29 mm). This is the second reason why the suture fiber content cannot be increased further. The higher the suture fiber content, the higher the pneumatic pressure required for 3D printing (Figure 3A). Increasing the pneumatic pressure causes more biomaterial ink to come out through the nozzle, which can increase the strand width. Therefore, a suture fiber content of 0.5% was the highest content that we were able to practically add in the gelatin biomaterial ink.

### 2.3. Printing Accuracy and Morphology of Suture Fiber Reinforced Gelatin 3D Scaffold

**Figure 4:**
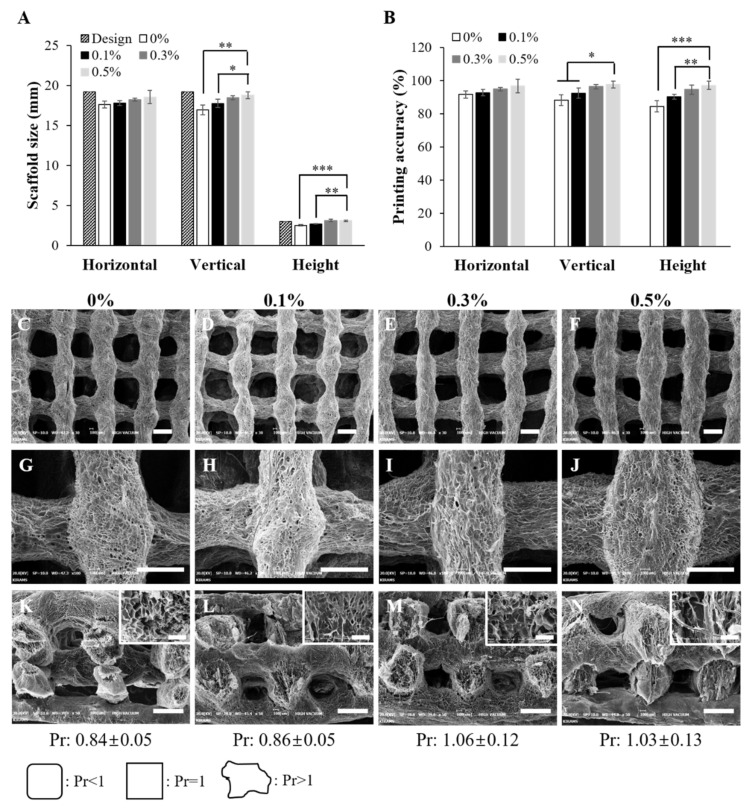
Printing accuracy, Pr value, and morphologies of the suture fiber reinforced gelatin 3D scaffold. (**A**) Plots of the sizes of the four different 3D printed scaffolds and of the CAD design. (**B**) Plots of the printing accuracies of the four different 3D printed scaffolds, with a 100% printing accuracy indicating no difference from the sizes of the CAD design (*n* = 4) (* *p* < 0.05, ** *p* < 0.01, and *** *p* < 0.001). (**C**–**N**) SEM images of the (**C**–**J**) surfaces and (**K**–**N**) cross-sections of the 3D printed scaffolds reinforced with suture fibers at contents of (**C**,**G**,**K**) 0%, (**D**,**H**,**L**) 0.1%, (**E**,**I**,**M**) 0.3%, and (**F**,**J**,**N**) 0.5% (scale bar: 500 μm; inset scale bar: 100 μm).

One of the greatest advantages of 3D printing technology is that we can fabricate sophisticated 3D scaffolds as designed. These advantages enable the fabrication of patient-specific scaffolds and desired disease models. With regards to printing accuracy of the biomaterial ink, synthetic polymer biomaterial inks (PEG, PVA, PLA, PLGA, and PCL) are superior to natural biomaterial inks [41,42]. However, synthetic polymer biomaterial inks are less biocompatible than natural polymer biomaterial inks, and the acidic by-products of their degradation can be toxic to surrounding cells [43]. In order to solve these problems, studies are needed to improve the printing accuracy of natural polymer biomaterial inks. Figure 4A,B shows a comparison of the sizes of the CAD model and the 3D printed scaffold. Observation of the printing accuracies for various contents of added suture fibers indicated a dramatic difference in the height of the 3D printed scaffold. Measurements of the heights of the 3D printed scaffolds showed that the use of a suture fiber content of 0.5% yielded a 3D printed scaffold with a printing accuracy of 97.1%, considerably higher than the 84.4% printing accuracy when without suture fibers. These results can be explained in terms of the modulus of the biomaterial inks (Figure 2C). When the modulus of the biomaterial ink was high enough for printing, the biomaterial ink maintained the round-type strand shape extending in a straight line when coming out through the nozzle. When the modulus of the biomaterial ink was not sufficiently high, the printed biomaterial ink could not maintain this shape and instead collapsed, with this collapse reducing the height of the 3D scaffold and consequently impairing the printing accuracy. These results show that the best printing accuracy and best printability were achieved by the printed biomaterial ink including a 0.5% suture fiber content, which also showed a dramatically increased modulus.

Another major advantage of 3D printing is the ability to use this technique to adjust the 3D scaffold pore sizes as desired. The presence of pores is very important for the human organ scale 3D scaffolds. A high interconnectivity of pores in the 3D scaffold can facilitate the supply of nutrients and oxygen to the cells in the 3D scaffold and increase the surface area of the 3D scaffold [44]. To achieve such interconnectivity, the pores at the surface area and those in the cross-sectional area must be well maintained. Figure 4C–N shows the surface and cross-section morphologies of the 3D printed scaffolds. The surfaces of the 3D printed scaffold strands were very rough and filled with micropores. Hydrogels with micropores and rough surfaces have already been reported to be better than hydrogels with smooth surfaces for tissue engineering [45]. The change in the 3D scaffold pore shape is also an important factor in 3D printing, so we evaluated the printability (Pr) value of each biomaterial ink. If the value of Pr is greater than 1, the shape of the pores is uneven and the uniformity of the strand is not good. Conversely, if value of Pr is lower than 1, the size of the pores is reduced compared to the CAD design and the size of the strands is increased. In Figure 4C–F, the Pr value was closest to 1 at 0.3% and 0.5%, which is consistent with the strand width (Figure 3C). The current 3D printed scaffolds were observed to have macropores, specifically with dimensions of 500–700 um. These macropores observed on the surfaces and in the cross-sections of the 3D printed scaffold could thus make it possible to supply nutrients and oxygen to the inside of organ scale 3D scaffolds.

### 2.4. Mechanical Strength of Suture Fiber Reinforced Gelatin 3D Scaffold

**Figure 5:**
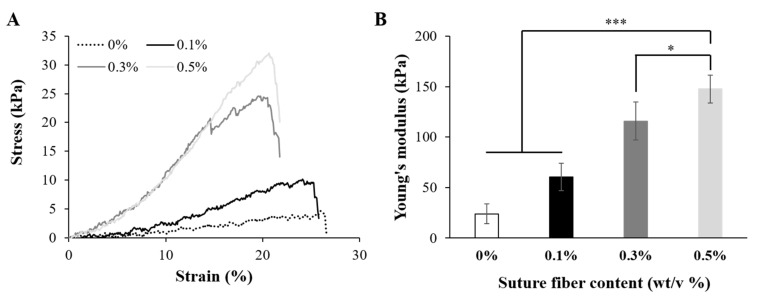
Mechanical properties of the 3D printed scaffolds (20 mm × 7 mm × 2 mm). (**A**) Tensile stress–strain curves of the 3D printed scaffolds with different suture fiber content. (**B**) Young’s modulus of the 3D printed scaffolds with different suture fiber content (*n* = 7) (* *p* < 0.05 and *** *p* < 0.001).

The mechanical strength of a 3D scaffold directly affects cell fate and tissue regeneration [46]. As reported by several groups, the modulus of native soft tissues and organs ranges from 0.1 kPa to 1000 kPa [25,47]. These reports showed that scaffolds for tissue engineering must have mechanical strength similar to those of the target tissues or organs in order to secure dimensional stability when exposed to surrounding human tissue movement and external stimuli. As shown in Figure 5, the Young’s modulus of the pure gelatin 3D printed scaffold was measured to be 24.0 ± 9.0 kPa. This low mechanical strength would limit the applicability of pure gelatin 3D printed scaffolds to only weak soft tissues such as brain, breast, and lung tissues. However, improving the mechanical strength of a gelatin hydrogel would allow it to be applied to muscle, skin, and tendon regeneration, especially since gelatin hydrogels already display an intrinsically high ability to be elongated. As shown in Figure 5A, the tensile stress of the 3D printed scaffold was markedly improved by increasing its suture fiber content. While increasing this suture fiber content was observed to be associated with a decrease in the elongation ratio, this effect was very slight. This result was probably due to the occurrence, during the 3D printing process, of a shear-induced alignment of the suture fibers as seen in the confocal microscopy images (Figure 7C–F). The significant differences between the tensile stress of the 3D printed scaffolds were also reflected in the considerable differences between their Young’s modulus. As shown in Figure 5B, the Young’s modulus of the 3D printed scaffold including a suture fiber content of 0.5% was, at 147.5 ± 14.0 kPa, about 6-fold higher than the 24.0 ± 9.0 kPa Young’s modulus of the 3D printed scaffold without suture fibers. As expected, the added suture fibers played an important role in improving the mechanical strength of the gelatin 3D scaffold.

### 2.5. Swelling Ratio of Suture Fiber Reinforced Gelatin 3D Scaffold

**Figure 6:**
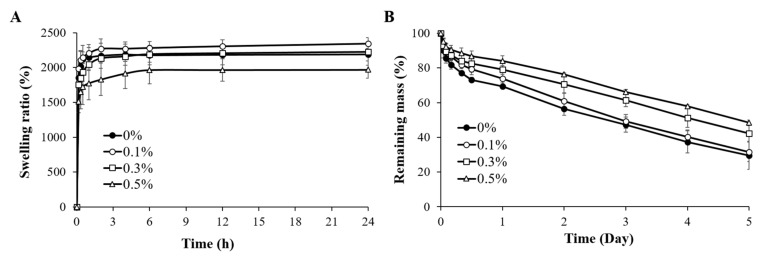
Physical properties of the suture fiber reinforced gelatin 3D scaffold. (**A**) Swelling ratios over time (*n* = 4). (**B**) Degradation of the suture fiber reinforced gelatin 3D scaffolds by 2 U/mL collagenase (*n* = 4).

The high water absorption capacity along with the excellent biocompatibility of natural polymers is one of the important reasons that natural polymers are suitable for use as biomaterial inks. Water-containing hydrophilic polymer materials are called hydrogels. Such hydrogels have cell-friendly properties such as facilitating rapid diffusion of nutrients, oxygen, and water-soluble compounds, providing protection from external stimuli, and improving cell adhesion and migration [19]. Therefore, water absorption is an essential property of the 3D scaffolds. In Figure 6A, the swelling ratio curves were acquired for the 3D printed scaffolds, in order to determine their ability to absorb water. For each 3D printed scaffold, the swelling ratio increased during the first 2 h to a value of approximately 2000% and plateaued stably at this level for the next 2 days of the swelling test. However, the swelling ratio of the 3D printed scaffold containing 0.5% suture fiber was, at 1969% at 24 h, slightly lower than those of the other scaffolds. This somewhat decreased ratio was due to its increased suture fiber content having resulted in a higher-density hydrogel structure, as can be inferred by its increased mechanical strength (Figure 5). Although there was a slight decrease in the swelling ratio due to the increase in the suture fiber content, it still exhibited a high swelling rate close to 2000%. The suture fiber reinforced 3D scaffold can deliver nutrients to culturing cells by rapidly absorbing liquid such as medium while maintaining their original structure.

### 2.6. Degradation Rate of Suture Fiber Reinforced Gelatin 3D Scaffold

3D scaffold implanted in vivo must be stable for enough time to provide an optimal environment for cell adhesion and proliferation [48]. Moreover, an overly rapid degradation of a scaffold can trigger an overly strong immune response, causing side effects at the transplant site [49]. Therefore, gelatin hydrogels have been subjected to various crosslinking methods to find ways to stop them from degrading rapidly during in vivo applications [28,29,30]. Physical crosslinking methods such as dried heat treatment (DHT) are more suitable for pure gelatin 3D scaffolds than chemical crosslinking methods [23]. However, suture fiber reinforced 3D scaffolds cannot be effectively crosslinked using the DHT method. DHT needs to be conducted at a high temperature (110 °C) and in a vacuum condition in which suture fibers denature and are unable to maintain their strength [50]. Accordingly, in the current work, suture fiber reinforced 3D scaffolds were crosslinked using a chemical reagent (EDC/NHS), and the rates of enzymatic degradation of these crosslinked scaffolds were confirmed by collagenase (2 U/mL). The rate of degradation of each 3D printed scaffold was determined by measuring the remaining mass of the scaffold as a function of time and plotting, as shown in Figure 6B, the remaining masses each as a percent of the original mass. At 5 days of the collagenase reaction, the remaining mass as a percentage of the original mass gradually increased with increasing suture fiber content. This remaining mass percentage for the 3D printed scaffold containing 0.5% suture fiber was approximately 48%, considerably higher than the approximately 29% remaining mass for the 3D printed scaffold without suture fibers. These results imply that supplementing suture fibers into a 3D printed scaffold could markedly improve the stability of the scaffold under physiological conditions.

### 2.7. Cell Proliferation Rate in Suture Fiber Reinforced Gelatin 3D Scaffold

**Figure 7:**
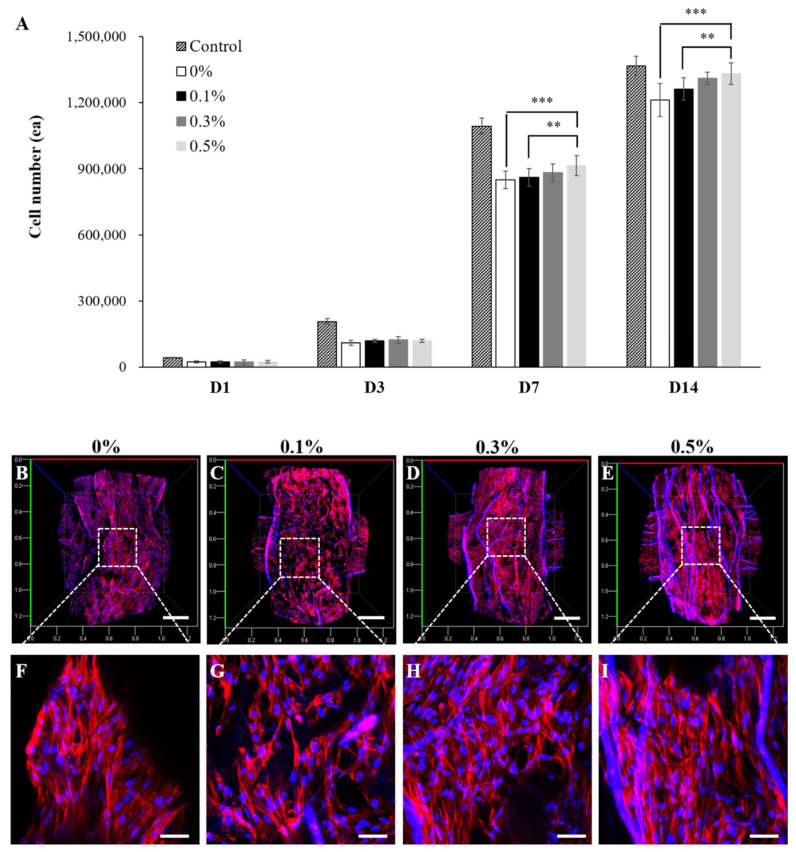
Biocompatibility of the suture fiber reinforced gelatin 3D scaffolds. (**A**) HDFs growth on each 3D printed scaffold over a culture time of up to 14 days (*n* = 4) (** *p* < 0.01, and *** *p* < 0.001). (**B**–**I**) Confocal microscopy images of HDFs at 14 days of being cultured on the 3D printed scaffolds prepared using suture fiber contents of (**B**,**F**) 0%, (**C**,**G**) 0.1%, (**D**,**H**) 0.3%, and (**E**,**I**) 0.5%. ((**B**–**E**) scale bar: 200 µm; (**F**–**I**) scale bar: 500 µm). The nuclei and F-actin of HDFs were stained with DAPI (blue) and phalloidin (red), respectively.

The biocompatibility of the 3D printed scaffolds was assessed by culturing HDFs on a 3D printed scaffold. The cell proliferation rate was evaluated based on an DNA assay. The morphologies and spreading of the cells were confirmed using confocal microscopy images. As shown in Figure 7A, all experimental groups supported steady cell growth for the duration of the cell culture. There was no significant difference in cell numbers between the experimental groups on day 1 and day 3. However, at 7 days and 14 days of cell culture, the cell growth rate tended to be higher for the cell cultured 3D scaffolds with a higher content of suture fibers. The number of HDFs increased rapidly from day 7 to day 14 of cell culture. At day 14, the DNA assay showed more cell proliferation having occurred on the cell cultured 3D scaffolds containing a higher suture fiber content. The most cells (1,331,707 ± 48,788 cells/scaffold) were observed for the cell cultured 3D scaffold containing 0.5% suture fiber. As shown in Figure 7B–I, the morphologies of the HDFs grown on the cell cultured 3D scaffold were visualized by performing confocal microscopy on samples in which the HDFs nuclei (DAPI, blue) and actin filaments (phalloidin, red) were stained. The confocal microscopy images taken at 14 days of cell culture showed that the HDFs were evenly distributed over the surfaces of the 3D printed scaffolds. Also, the number of DAPI-stained HDFs present on the 3D printed scaffold containing 0.5% suture fiber was higher than those on the other experimental groups. These results, which are consistent with the results of the DNA assays shown in Figure 7A, may have been due to the obvious interconnected and hierarchically structured pores and good dimensional stability of the 3D printed scaffold containing 0.5% suture fiber (as shown in Figure 4). Such structural and dimensional stability features of 3D printed scaffolds play an important role in cellular behaviors such as cell proliferation, migration, infiltration, and morphogenesis because they affect the cellular microenvironment [51]—but these features may also be influenced by cell-mediated contraction.

### 2.8. Dimensional Stability of Cell Cultured 3D Scaffolds

**Figure 8:**
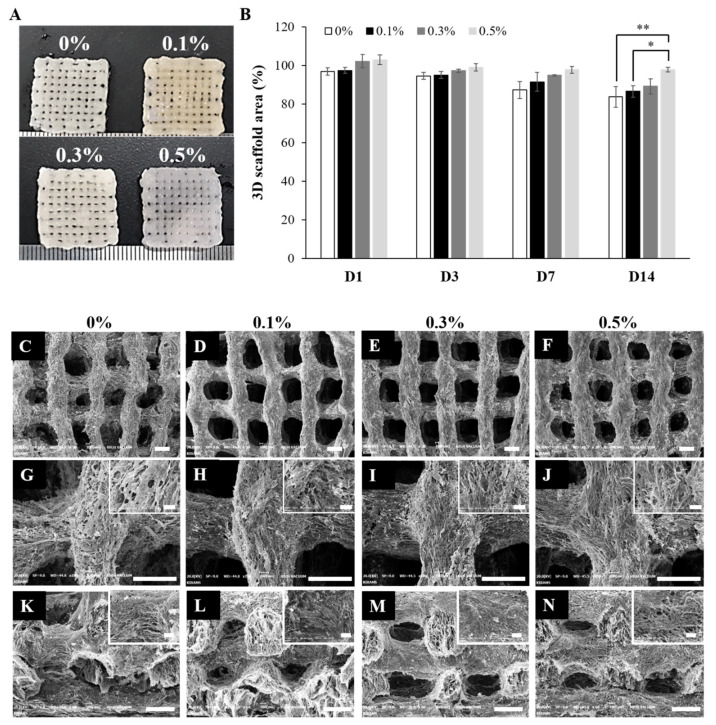
Effect of suture fiber content on 3D printed scaffold cell-mediated contraction. (**A**) Images of the cell cultured 3D scaffolds at 14 days of culture. (**B**) Cell cultured 3D scaffold areas (%) at different culture times (*n* = 4) (* *p* < 0.05 and ** *p* < 0.01). (**C**–**N**) SEM images at 14 days of culture of the (**C**–**J**) surfaces and (**K**–**N**) cross sections of the cell cultured 3D scaffolds reinforced with suture fibers at contents of (**C**,**G**,**K**) 0%, (**D**,**H**,**L**) 0.1%, (**E**,**I**,**M**) 0.3%, and (**F**,**J**,**N**) 0.5%. (Scale bar: 500 μm; inset scale bar: 100 μm.)

Contractile forces generated by cells are one of the important factors that impair the dimensional stability of a scaffold. B. A. Harley et al. reported a contractile force of about 11~41 nN generated by dermal fibroblast in a collagen-glycosaminoglycan (GAG) scaffold [52]. M. Eastwood et al. reported that a 0.1 nN/cell contractile force occurred in a collagen hydrogel [53]. The cell-mediated contractile force affects the cellular microenvironment by changing the size and microstructure of the 3D scaffold. These changes in the size of the 3D scaffold cause the implanted 3D scaffold to separate from the surrounding natural tissue. In addition, changes in 3D scaffold microstructure features such as pore size and porosity caused by cell-mediated contraction affect cell migration, infiltration, and nutrient exchange. Therefore, it is important to maintain the dimensional stability of the 3D scaffold by minimizing cell-mediated contraction. As shown in the digital images in Figure 8A, at day 14 of cell culture, the sizes of the cell cultured 3D scaffolds containing a low content of suture fibers were smaller than those of the scaffolds with a high suture fiber content. As shown in Figure 8B, the areas and thicknesses of the cell cultured 3D scaffolds were measured at several time points, and the contraction of each cell cultured 3D scaffold was indicated by showing the decrease in the cell cultured 3D scaffold area (%) over 14 days. At 14 days of cell culture, the area of the cell cultured 3D scaffold containing 0.5% suture fiber was 97.8% of the initial area, quite a bit higher than the 83.8% of the initial area maintained by the cell cultured 3D scaffold without suture fibers. As shown in Figure 8C–N, this difference was also confirmed upon inspection of the corresponding SEM images. It was confirmed that numerous pores present on the strand surfaces of all the cell cultured 3D scaffolds were not visible by cells and ECM at 14 days of cell culture. As shown in Figure 8C–J, the strand morphology was changed by the proliferated cells. In the cell cultured 3D scaffolds containing 0% and 0.1% suture fiber, the strand size was reduced, the strand uniformity decreased, and, as shown in Figure 8K,L, the pores collapsed with regards to their cross-section areas; on the other hand, the cell cultured 3D scaffolds containing 0.3% and 0.5% suture fiber had uniform strands and maintained the pore structure in the cross-section images of Figure 8E,F,I,J,M,N. To reduce cell-mediated contraction, several studies have adjusted the gel strength by increasing the degree of crosslinking or the concentration of matrix substances or introduced GAG. [54,55]. We have now also provided compelling evidence that introducing suture fibers could contribute to the dimensional stability of 3D scaffolds and increase the mechanical strength of these scaffolds so that they can resist cell-mediated contraction.

### 2.9. Mechanical Strength of Cell Cultured 3D Scaffolds

**Figure 9:**
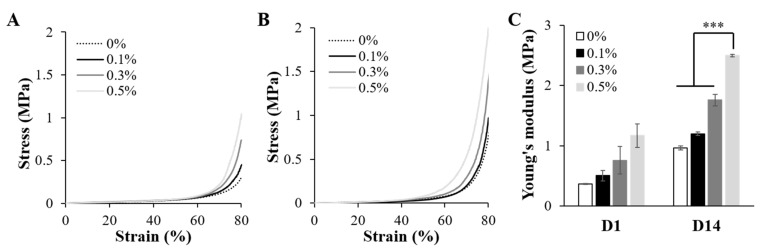
Mechanical properties of the cell cultured 3D scaffolds. (**A**,**B**) Compressive stress–strain curves of the cell cultured 3D scaffolds at (**A**) 1 day and (**B**) 14 days of culture. (**C**) Young’s modulus of the cell cultured 3D scaffolds (*n* = 4) (*** *p* < 0.001).

The Young’s modulus of the 3D printed scaffold containing 0.5% suture fiber, while 6 times the modulus of the 3D printed scaffold without suture fibers (Figure 5), was still not as high as the Young’s modulus of human skin, and hence this scaffold cannot be used to replace human skin. Nevertheless, a higher modulus can be obtained by carrying out cell proliferation and an ECM remodeling process. D. Kang et al. mixed HepG2/C3A/endothelial cells with collagen/alginate bioink and printed them in order to mimic the hepatic lobule structure [56]. They showed that the compressive strength of the printed hepatic lobe was about 2 times higher on day 5 than on day 1. The mechanical strength of the suture fiber reinforced 3D scaffolds used in this study also increased with cell culture time. As shown in Figure 9A,B, the compressive stress of each cell cultured 3D scaffold was higher on day 14 of the cell culture than on day 1. Moreover, as shown in Figure 9C, the Young’s modulus of each cell cultured 3D scaffold on day 14 was more than twice the Young’s modulus on day 1. The Young’s modulus of the cell cultured 3D scaffold containing 0.5% suture fiber was 2.496 ± 0.019 MPa at 14 days of cell culture. The improvements in the mechanical strengths of the 3D printed scaffolds with cell culture time were probably due to the proliferation of cells and ECM secreted from cells (Figure 8C–N). This increase in mechanical strength and resistance to cell-mediated contraction indicated that a suture fiber reinforced 3D scaffold with high dimensional stability and mechanical strength can stably maintain its structure for a sufficient amount of time in a physiological environment.

## 3. Materials and Methods

### 3.1. Preparation of the Biomaterial Inks

Gelatin biomaterial inks containing suture fibers were prepared by adding suture fibers into a gelatin aqueous solution. Briefly, the suture bundles (Vicryl, Ethicon, Somerville, NJ, USA) were cut with surgical blades into filaments each having a length of 3 mm. By using a mortar, the bundles were formed into suture fibers. Gelatin (Bloom 225, type A, MP Biomedicals, Solon, OH, USA) was mixed with distilled water (DIW) at 70 °C for 3 h to form a solution with a final gelatin concentration of 3.5% (*w*/*v*) [23]. The suture fibers and 3.5% gelatin aqueous solution were then mixed at room temperature. Various biomaterial ink formulations containing suture fibers at content of 0% (*w*/*v*), 0.1% (*w*/*v*), 0.3% (*w*/*v*), and 0.5% (*w*/*v*), respectively, were prepared. Inclusion of suture fibers in the biomaterial inks was confirmed using scanning electron microscopy (SEM) (CX-200TM; COXEM, Daejeon, Korea). Briefly, the gelatin biomaterial inks containing suture fibers were transferred to a −80 °C deep freezer (MDF-U74V, SANYO, Osaka, Japan) and frozen overnight. The frozen samples were dried by freeze-drying for 3 days. The dried samples were coated with gold using an ion sputter coater (SPT-20; COXEM, Daejeon, Korea) and characterized using SEM at 20 kV.

### 3.2. Rheological Properties of the Biomaterial Inks

The rheological properties of the suture fibers added gelatin biomaterial inks were confirmed using an AR 2000ex rheometer (TA instruments, New Castle, DE, USA) with a cone–plate geometry (diameter of 40 mm, angle of 1°). All samples were heated to 37 °C before being used in the experiment. The viscosities of the samples were measured as a function of shear rate, with the shear rate increased from 0.1 to 10/s at 37 °C. Viscosity measurements were also taken at various temperatures, starting at 40 °C and then decreasing at a cooling rate of 1.0 °C/min to 10 °C. Here, the oscillations were applied at a frequency of 1 Hz, and the shear rate was 0.1/s. The storage modulus (*G*′) and loss modulus (*G*″) of each biomaterial ink was also determined, by placing the biomaterial ink on the rheometer plate and lowering the temperature from 37 °C to 20 °C in 30 min. Then, frequency sweep experiments were conducted at a fixed strain of 1% from 0.1 to 10 Hz at 20 °C. The complex shear modulus (G*) was calculated using Equation (1) [57]:

(1) $$\mathrm{Complex}\;\mathrm{shear}\;\mathrm{modulus}\;\left(G*\right)=\sqrt{{\left(G^{\prime }\right)}^{2}+{\left(G^{\prime \prime }\right)}^{2}}$$

### 3.3. Fabrication of 3D Scaffolds

A low-temperature 3D printing system was built by modifying a commercial 3D printer [23]. The modified 3D printer was driven using a customized operating program (MotionMaster) (KIRAMS, Seoul, Korea). All 3D scaffolds, except those used for taking measurements of mechanical properties, were designed (Solidworks) to have dimensions of 19.2 mm × 19.2 mm × 3 mm (5 layers). During 3D printing, the temperature of the internal environment was maintained at 20–22 °C, and the cryogenic plate temperature was kept at −10 °C. After transferring 8 mL of a biomaterial ink to a 10 mL syringe, the rubber plunger of the syringe was replaced with a PLF-E plunger (MUSASHI, Mitaka, Japan). Three-dimensional printing was carried out while changing the 3D printing parameters to evaluate biomaterial ink printability, with pneumatic pressures of 20–80 kPa and printing speeds of 5–10 mm/s. The needle size was fixed at 18 gauge (inner diameter: 0.84 mm). A porous 3D scaffold was obtained as each layer adhered to the previous layer to form a 0°/90° strand structure. The printed strand width was confirmed by using vernier calipers (Mitutoyo, Kawasaki, Japan). Immediately after carrying out the 3D printing, the 3D scaffolds were transferred to a −80 °C deep freezer and frozen overnight. Then, the 3D scaffolds were dried by freeze-drying for 24 h. The lyophilized 3D scaffolds were stored at −20 °C, to prevent them from absorbing water, until they were used for later experiments.

### 3.4. Crosslinking of 3D Scaffolds

The 3D scaffolds were crosslinked using a chemical crosslinking agent. An amount of 10 mM of 1-ethyl-3-(3-dimethylaminopropyl)-carbodiimide hydrochloride (EDC) (Daejung, Siheung, Korea)/5 mM of N-hydroxysuccinimide (NHS) (Sigma, Saint Louis, MO, USA) was dissolved in a 90% aqueous ethanol solution. Samples of the lyophilized 3D scaffolds were placed in a 6-well plate, and a 10 mM EDC/5 mM NHS solution was added to each well at room temperature. After 24 h, the crosslinked 3D scaffolds (3D printed scaffolds) were washed three times with 90% ethanol. The 3D printed scaffolds were immersed in a phosphate-buffered saline (PBS) (Welgene, Gyeongsan, Korea) solution.

### 3.5. Morphology and Printing Accuracy of Suture Fiber Reinforced Gelatin 3D Scaffold

The printing accuracy of the 3D printed scaffolds was compared with the CAD design by applying the method proposed by M.D. Giuseppe et al. [58]. The size *Di* (horizontal, vertical, and height) of the 3D printed scaffolds were measured using vernier calipers. The printing accuracy (%) was determined according to Equation (2):

(2) $$\mathrm{Printing}\;\mathrm{accuracy}\;\left(\%\right)=\left[1-\frac{\left|Di-D\right|}{D}\right]\times 100$$

where *Di* and *D* represent the 3D printed scaffold size and CAD designed size, respectively.

The surface and cross-section morphologies of the 3D printed scaffolds were visualized using SEM. The 3D printed scaffolds were transferred to a −80 °C deep freezer and frozen overnight. Then, the 3D printed scaffolds were dried by freeze-drying for 24 h. The 3D printed 3D scaffolds were coated with gold using an ion sputter coater and were characterized using SEM at 20 kV. The printability (*Pr*) value is highly correlated with the circularity (*C*) of the strand. The circularity of a strand is defined as in Equation (3):

(3) $$C=\frac{4\pi A}{L^{2}}$$

where *L* and *A* represent the mean perimeter and mean area, respectively. If the *C* value is 1, the strand shape is a complete circle shape. In a square shape, circularity is equal to π/4. To determine the *Pr* value of each biomaterial ink, we defined the *Pr* based on a square shape using Equation (4) [59]:

(4) $$\mathrm{Pr}=\frac{\pi }{4}\times \frac{1}{C}=\frac{L^{2}}{16A}$$

SEM images of 3D printed scaffolds were analyzed in ImageJ software to determine the perimeter and area of pores (*n* = 4).

### 3.6. Physicochemical Properties of Suture Fiber Reinforced Gelatin 3D Scaffold

The mechanical properties of the 3D printed scaffolds were determined using a universal testing machine (Instron 5960; Instron^®^, Norwood, MA, USA). For the tensile strength test, 3D scaffolds (20 mm × 7 mm × 2 mm) were fabricated and crosslinked. The 3D printed scaffolds were immersed in a PBS solution at 37 °C for 1 h, and the experiment was conducted. The data used to construct the tensile stress–strain curves of the 3D printed scaffolds were collected using a stretching speed of 10 mm/min. The experiment was stopped at the point where the sample broke. The compressive strength test was performed using cell cultured 3D scaffolds (19.2 mm × 19.2 mm × 3 mm). After 1 day and 14 days of culture, the cell cultured 3D scaffolds were washed three times with Dulbecco’s phosphate-buffered saline (DPBS) (Welgene, Gyeongsan, Korea) solutions and fixed with 4% paraformaldehyde (Sigma, Saint Louis, USA) solutions at 4 °C overnight. The fixed cell cultured 3D scaffolds were washed three times with PBS solutions. The experiments were each conducted with a crosshead speed of 3 mm/min at room temperature. The point where the strain rate became 80% was set as the end point of each experiment. For each sample, the Young’s modulus of the tensile and compressive test was calculated from the stress–strain curve.

The swelling ratios of the 3D printed scaffolds were determined from measurements of changes in their weights. First, the 3D printed scaffolds were transferred to a −80 °C deep freezer and frozen overnight. They were then dried by freeze-drying for 24 h. After being lyophilized, the weight of each dried 3D printed scaffolds was measured ($W_{0}$). Then, the dried 3D printed scaffolds were immersed in a PBS solution at 37 °C. The weight of each 3D printed scaffold was measured at desired time intervals (10, 20, 30, 60, 120, 240, 360, 720, and 1440 min). The swelling ratio (%) was calculated using Equation (5) [60]:

(5) $$\mathrm{Swelling}\;\mathrm{ratio}\;\left(\%\right)=\frac{W_{t}-W_{0}}{W_{0}}\times 100$$

Here, for each 3D printed scaffold sample, $W_{t}$ and $W_{0}$ represent the weight of the immersed sample and that of the dried sample, respectively.

The in vitro degradability of each 3D printed scaffold was confirmed by using collagenase. Each dried 3D printed scaffold was immersed in a PBS solution at 37 °C for 1 h ($W_{o}$) and then transferred to a PBS solution containing collagenase (Type I, 2U/mL, Sigma) at 37 °C. The experiment was conducted for 5 days, and the weight of the sample was measured at each time point ($W_{t}$). The remaining mass (%) was calculated using Equation (6) [61]:

(6) $$\mathrm{Remaining}\;\mathrm{mass}\;\left(\%\right)=\frac{W_{t}}{W_{o}}\times 100$$

Here, for each 3D printed scaffold sample,$\;W_{t}$ and $W_{0}$ represent the weight of the sample after incubation and that of the original sample, respectively.

### 3.7. Cell Culture and Seeding on the 3D Scaffolds

Human dermal fibroblast (HDF) (American Type Culture Collection (ATCC), Manassas, USA) cells were cultured in Dulbecco’s Modified Eagle’s Medium (DMEM) (Gibco, Amarillo, TX, USA) supplemented with 10% fetal bovine serum (FBS) (Tissue Culture Biologicals, Long Beach, CA, USA) and 1% penicillin/streptomycin (Welgene, Gyeongsan, Korea). For each experiment, the medium was replaced every other day. After reaching 70–80% confluency, the cells were detached by using trypsin-EDTA and viable cells were counted using a trypan blue assay. To evaluate the cell affinities of the 3D printed scaffolds, the HDFs were cultured on the 3D printed scaffolds. Before being seeded with the cells, these scaffolds were sterilized with 70% ethanol and ultraviolet light for over 12 h. The sterilized 3D printed scaffolds were then washed three times with DPBS, transferred to a 6-well plate (ultra-low attachment surface, Corning, Corning, USA), and then immersed in DMEM for 24 h for medium pre-wetting. After the pre-wetting, the medium was removed from the 6-well plate. The suspensions of HDFs (*p* = 9, 3 × 10^4^ cells/100 μL/scaffold) were seeded on the sterilized 3D printed scaffolds and incubated at 37 °C with 5% CO_2_ for 3 h. After the 3 h incubation, 5 mL of DMEM was added to each well and the medium was changed every second day. Cells were cultivated at 37 °C, 5% CO_2_ for 1, 3, 7, or 14 days.

### 3.8. Cell Proliferation

Cell proliferation was evaluated using a DNA assay. The DNA assay (Quant-iT^™^ Picogreen^™^ dsDNA Assay kit, Invitrogen, Waltham, MA, USA) was used for cell number confirmation. After the desired culture time, the HDF numbers were evaluated according to the manufacturer’s protocol. To quantify the amount of DNA, the fluorescence intensity was measured using a microplate reader (SpectraMax M2e; Molecular Devices, San Jose, CA, USA) at an excitation wavelength of 480 nm and an emission wavelength of 560 nm.

### 3.9. Cell Morphology

The morphologies of the HDFs on the 3D printed scaffolds were visualized using SEM and confocal microscopy (LSM 710; Carl Zeiss, Oberkochen, Germany). After 14 days of culture, the cell cultured 3D scaffolds were washed three times with DPBS and fixed with 4% paraformaldehyde solution. The fixed cell cultured 3D scaffolds were washed three times with PBS solutions, and then frozen at −80 °C (overnight) and lyophilized for 24 h. The dried samples were coated with gold and imaged using SEM at 20 kV. To obtain confocal images, the fixed specimens were treated with 0.3% Triton-X for 30 min to permeate the cell membrane. Phalloidin (Alexa 594-phalloidin; Molecular probes, Eugene, USA) was used for F-actin staining and DAPI (D9542; Sigma, Saint Louis, USA) for nuclear staining. After the stainings, the HDFs were visualized using a laser scanning confocal microscope system.

### 3.10. Cell-Mediated Contraction of the Cell Cultured 3D Scaffold

Cell-mediated contractions of the 3D printed scaffolds, specifically mediated by the HDFs, were evaluated. After the desired culture time, the horizontal, vertical, and height dimensions of the cell cultured 3D scaffolds were measured using vernier calipers. They were graphed as a percentage of the cell cultured 3D scaffold area versus day 0. The cell-mediated contraction in each case was calculated using Equation (7) [51]:

(7) $$\mathrm{Scaffold}\;\mathrm{area}\;\left(\%\right)=\frac{A_{t}}{A_{o}}\times 100$$

where $A_{t}$ and $A_{0}$ represent the 3D printed scaffold areas after the desired time point and at day 0, respectively.

### 3.11. Statistical Analysis

All experiments were independently repeated at least four times, and data are presented with the mean standard error. Statistical analyses were performed using the Student’s *t*-test. The significance levels were set at * *p* < 0.05, ** *p* < 0.01, and *** *p* < 0.001.

## 4. Conclusions

We fabricated suture fiber reinforced gelatin 3D scaffolds using 3D printing technology. The suture fibers improved the printability and printing accuracy of the gelatin biomaterial ink by increasing its rheological properties. Three-dimensional scaffolds have high pore interconnectivity and a distinct hierarchical structure after crosslinking. Supplementing suture fibers at a 0.5% content in the gelatin biomaterial ink significantly improved the mechanical strength of the 3D printed scaffold. This increase in mechanical strength showed the possibility that the suture fiber reinforced gelatin 3D scaffold can be applied to skin, muscles, and tendons that require higher mechanical strength in addition to soft tissues such as the brain, lungs, and liver. In cell studies aimed to confirm the biocompatibility of the suture fiber reinforced gelatin 3D scaffolds, the 3D printed scaffold containing 0.5% suture fibers supported the best HDFs proliferation rate and cell spreading. In particular, the results showing resistance to cell-mediated contraction and improved mechanical strength demonstrated that the supplement of the suture fibers could maintain the dimensional stability of the 3D scaffold by preventing the collapse of the 3D structure during the cell culture. These results show that the suture fiber reinforced gelatin 3D scaffolds can be applied to more diverse organs as patient-specific tissue scaffolds.

## Data Availability

All results generated or analyzed during the present study are included in this published article. Data and materials can be made available from the corresponding author (chkim@kcch.re.kr) upon reasonable request.
